# Predicting trend of early childhood caries in mainland China: a combined meta-analytic and mathematical modelling approach based on epidemiological surveys

**DOI:** 10.1038/s41598-017-06626-w

**Published:** 2017-07-26

**Authors:** Xiaonan Zhang, Lei Zhang, Yonghong Zhang, Zhaoying Liao, Jinlin Song

**Affiliations:** 1 College of Stomatology, Chongqing Medical University, Chongqing, China; 2 Chongqing key Laboratory of Oral Diseases and Biomedical Sciences, Chongqing, China; 3 Chongqing Municipal Key Laboratory of Oral Biomedical Engineering of Higher Education, Chongqing, China; 4 Melbourne Sexual Health Centre, Alfred Health, Melbourne, VIC Australia; 5 Central Clinical School, Faculty of Medicine, Nursing and Health Sciences, Monash University, Melbourne, VIC Australia; 6 Research Center for Public Health, School of Medicine, Tsinghua University, Beijing, China; 7 Medicine Engineering Research Center, College of Pharmacy, Chongqing Medical University, Chongqing, China; 8 Children’s Hospital of Chongqing Medical University, Chongqing, China

## Abstract

Early childhood caries (ECC) is the most common chronic disease in young children. A reliable predictive model for ECC prevalence is needed in China as a decision supportive tool for planning health resources. In this study, we first established the autoregressive integrated moving average (ARIMA) model and grey predictive model (GM) based on the estimated national prevalence of ECC with meta-analysis from the published articles. The pooled data from 1988 to 2010 were used to establish the model, while the data from 2011 to 2013 were used to validate the models. The fitting and prediction accuracy of the two models were evaluated by mean absolute error (MAE) and mean absolute percentage error (MAPE). Then, we forecasted the annual prevalence from 2014 to 2018, which was 55.8%, 53.5%, 54.0%, 52.9%, 51.2% by ARIMA model and 52.8%, 52.0%, 51.2%, 50.4%, 49.6% by GM. The declining trend in ECC prevalence may be attributed to the socioeconomic developments and improved public health service in China. In conclusion, both ARIMA and GM models can be well applied to forecast and analyze the trend of ECC; the fitting and testing errors generated by the ARIMA model were lower than those obtained from GM.

## Introduction

The early childhood caries (ECC), the tooth decay occurred in any primary tooth in a child 71 months of age or younger^1^, has been reported as the most prevalent infectious disease of children. The ECC prevalence in mainland China is comparatively high, 65.5% for 1–6-year-olds and 66.1% for 5-year-olds^2^, far from the target by WHO “half of the 6-year-old children are caries-free”^3^. It has an adverse impact not only on children’s nutrition intake, speech, and daily routine activities, but also on their physiological health^4, 5^. China is a rapidly developing country with the largest children population in the world^6^. Children’s oral health has become a major public health problem in China. Hence, to better aid an explicit and quantitative direction for the future oral health plan among these population, a reliable prediction method to understand the trend of ECC is needed.

Forecasting techniques, which have been extensively applied to analyze the occurrences, development, and future trends of diseases, such as tuberculosis^7^, malaria^8^, hepatitis^9^, diabetics^10^ and influenza^11^, serve as a policy-supportive tool effectively. The first step to establish the forecasting models was to acquire the time sequence^12^. Traditionally, the data used for forecasting came from the regional reports or surveillance data. However, we can hardly get the series data of ECC prevalence by year. Since 1980’s, the Chinese Ministry of Health has invested large human and financial resources to conduct national oral epidemiology survey every decade. The first national survey was conducted in 1982, and the surveyed population were mainly students from primary and secondary schools. In the second and third national oral surveys in 1995 and 2005^13, 14^, 11 and 30 provinces were covered respectively, and children aged 5 were chosen as a representative age group. The surveys focused on levels of caries, periodontal disease, mucosal disease and dental fluorosis. Since then, no national surveys on ECC have been performed in China. So far there is no publication devoted to the prediction of ECC, specifically on the national level.

To do the point prediction, the time series analysis is the most commonly used method in statistic. Autoregressive integrated moving average (ARIMA) model^15^ is one of the common means of the time series analysis with a complete theoretical basis, which can provide middle-long term forecast analysis. Comparing to other statistical models, the characteristic of the grey predictive model (GM) is outstanding^16, 17^. It only needs a small sample to establish the model and to predict with a certain precision, which is especially applicable for the system with fuzzy structure or imperfect data. Therefore, these two models are applicable in this study for the prediction.

To establish the optimal model to predicate the trend of ECC in mainland China, we pooled data from existing reports with meta-analysis to calculate the national prevalence of ECC from 1988 to 2013. Then, we forecasted ECC prevalence in mainland China from 2014 to 2018 by the established ARIMA and GM models. The result was expected to provide quantitative basis for allocating medical resources to prevent and control ECC.

## Materials and Methods

### Data sources

Data used for establishing ARIMA and GM models came from the combined results of a meta-analysis, which was conducted according to the preferred reporting items for systematic review and meta-analyses (PRISMA) checklist. This approach has already been published in the previous literature^2^. No ethical statement was necessary because all data were secondary summary data.

Peer-reviewed articles were searched in the following databases from the date of establishment to March, 2016: PubMed, Embase, Chinese Biomedical Literature database (CBM), Chinese National Knowledge Infrastructure database (CNKI), Chinese Wan Fang database, and Chongqing VIP database, using the key terms ‘caries’, ‘prevalence’, ‘epidemiology’, and ‘China’. Two authors screened articles and extracted data independently. Any disagreement was resolved by consensus or the third author. A manual search was also applied to the relevant reference lists of all the eligible articles. Studies were included if they were cross-sectional surveys on ECC using random sampling, at city-level or above in mainland China (except for Hong Kong, Taiwan, and Macao). In order to exclude the effect of age structure, 5-year-olds were chosen as a representative age group. Additionally, studies were based on the general population rather than a specific group. The language of studies was limited to English and Chinese.

To reflect the temporal distribution of ECC, prevalence estimates for ECC in 5-year-olds in each survey year (1988–2013) were calculated by pooling the data from each study, with STATA software 11.1 (Stata, College Station, TX, USA). Statistical heterogeneity was detected by Q-test and I^2^-statistics. A random effects model was adopted in the case of significant heterogeneity (I^2^ > 50% or *P* < 0.1). The quality of the selected studies was assessed using the Reporting of Observational Studies in Epidemiology (STROBE) guideline (Table S2)^18^. Potential publication bias was evaluated by funnel plots and Begg’s test; *P* ≤ 0.05 was considered to be significant.

Combined prevalence rates were divided into two parts to compare the fitting and prediction performances: data from 1988 to 2010 were used to construct the models, while data from 2011 to 2013 were used to test the prediction accuracy of the models. Chow breakpoint test^19^ was adopted to identify whether there had been a structural change around 2010; the result was regarded to be significant if *P* ≤ 0.05.

### ARIMA model construction

ARIMA is a traditional method to study the time series data. Since the sequence of ECC prevalence is a time series and generally have a trend, we chose ARIMA (p, d, q) model to fit it. The following parameters were selected when fitting the ARIMA model: p, the order of auto-regression; d, the degree of difference; q, the order of moving average^12, 20^.

The sequence of prevalence usually had a trend which was non-stationary, thus augmented dickey-fuller unit root (ADF) test and KPSS test were chosen to test the stationary of the original sequence. If the sequence was non-stationary, differencing was used to transform it to stationary sequence. Under the circumstance, d = 1. And the tests were made on the differenced sequence to identify whether the trend still existed. If “yes”, d = 2, and process went on until the sequence was stationary. Generally, when d = 2, the process could stop^21^.

When the differenced sequence was stationary, the variance and covariance of the sequence did not change over time. Then the autocorrelation function (ACF) graph and partial autocorrelation function (PACF) graph were used to identify the order of auto-regression (AR) and the order of moving average (MA) in the ARIMA model^22^. The model was fitted by the least squares method. The t-statistic was used to test the significance of the parameters and the F-statistic was used to test the significance of the equation. In addition, the Akaike Information Criterion (AIC) was certainly considered to be a comprehensive identifier of the parameters, and the R-squared (R^2^) was an important index for model testing^23^.

**Figure 1:**
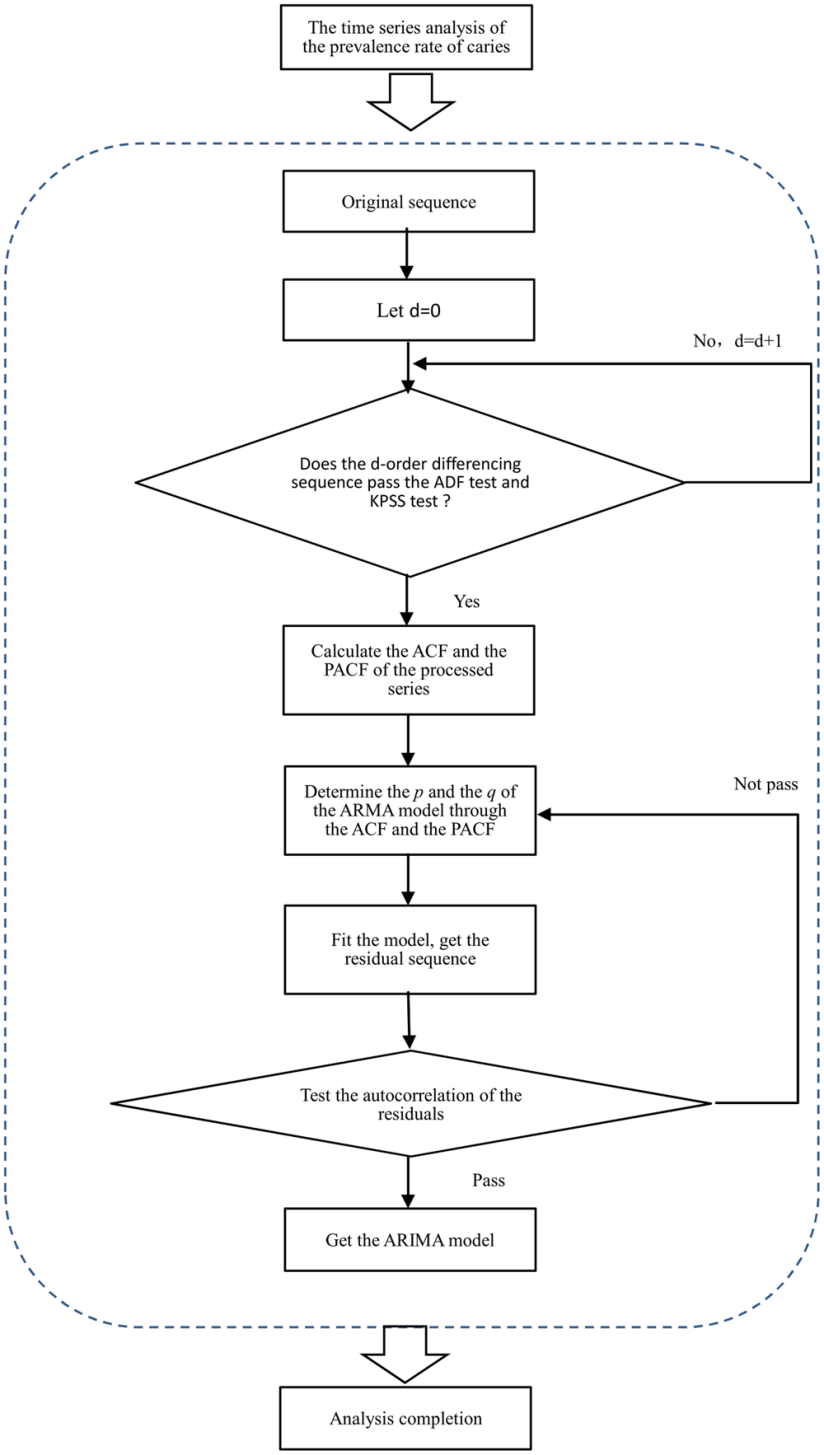
Flow chart to construct the ARIMA model.

At last, the residual series would be analysed by the Ljung-Box Q-test^24^ to verify whether it was a white noise time series or not. The white noise series would indicate that the information has been sufficiently extracted, allowing the model to conduct the predictive analysis. Otherwise, the order re-determination and parameter re-estimation were needed. We used the obtained model to forecast the prevalence of ECC from 2011 to 2018. The flow chart to construct ARIMA model was illustrated in Fig. 1.

### GM (1,1) construction

If a system is fuzzy in hierarchy relationship, random in dynamic change, and uncertainty in indicator databases, the system is called grey system. The modeling for a grey system is grey model. GM (1,1) is the typical representative for the grey model, which can be used to fit and forecast in the complex system.

Firstly, an accumulative sequence had to be made on the original sequence. Then the new sequence was assumed to be adopted to the differential equation as follows:

1 $$\frac{dy(t)}{dt}+\alpha y(t)=\mu $$

Finally, solving the equation, the GM(1,1) was constructed^16^. We also used the established grey model to forecast the future prevalence of ECC from 2011 to 2018.

### Performance Statistics Index

The ARIMA model was created with EVIEWS 8 with a significant level of *P* < 0.05; the GM (1,1) was constructed with Matlab 7.0. In order to compare the performance, two statistics indexes were used to evaluate the fitting and prediction accuracy: the mean absolute error (MAE) and the mean absolute percentage error (MAPE). Their calculation formulas were as follows:

2 $$MAE=\frac{1}{n}\sum _{t=1}^{n}|{y}_{t}-{\hat{y}}_{t}|$$

3 $$MAPE=\frac{1}{n}\sum _{t=1}^{n}\frac{|{y}_{t}-{\hat{y}}_{t}|}{{y}_{t}}$$

where *y*_*t*_ and ${\hat{y}}_{t}$ denote the original and the predicted value respectively at time t. The smaller these two indexes were, the better the fitness and prediction performances.

## Results

### Results from meta-analyses

#### Literature search and quality assessment

A total of 11,776 publications were identified, and 78 eligible articles were included in the meta-analysis (Supplementary Fig. S1). The characteristics of the 78 articles were summarized in Supplementary Table S1. Quality assessment showed that all the studies scored at least 7 out of 10. Publication bias was statistically significant (Begg’s test, *P* < 0.001).

#### Temporal trends in prevalence of ECC

**Figure 2:**
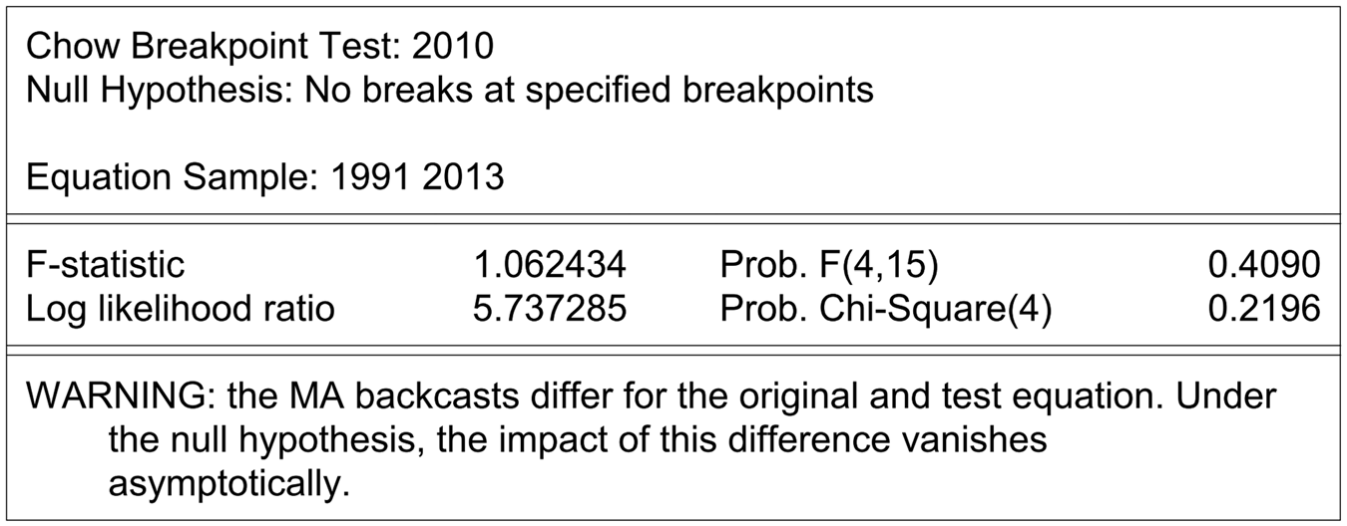
The result of chow breakpoint test.

The pooled prevalence of ECC among 5-year-olds was 66.1% (95% CI: 59.0–73.4%, ranging from 81.2% in 1988 to 56.1% in 2013. Figure 2 illustrated the trends in the prevalence at age 5 over time and a decreasing trend in ECC prevalence was observed during the study period.

The result of chow breakpoint test indicated that no structural change occurred in 2010 (*P* > 5%, Fig. 2). In other words, there was no deviation between the fitting data and the forecasting data.

### Simulation Results

#### ARIMA

**Figure 3:**
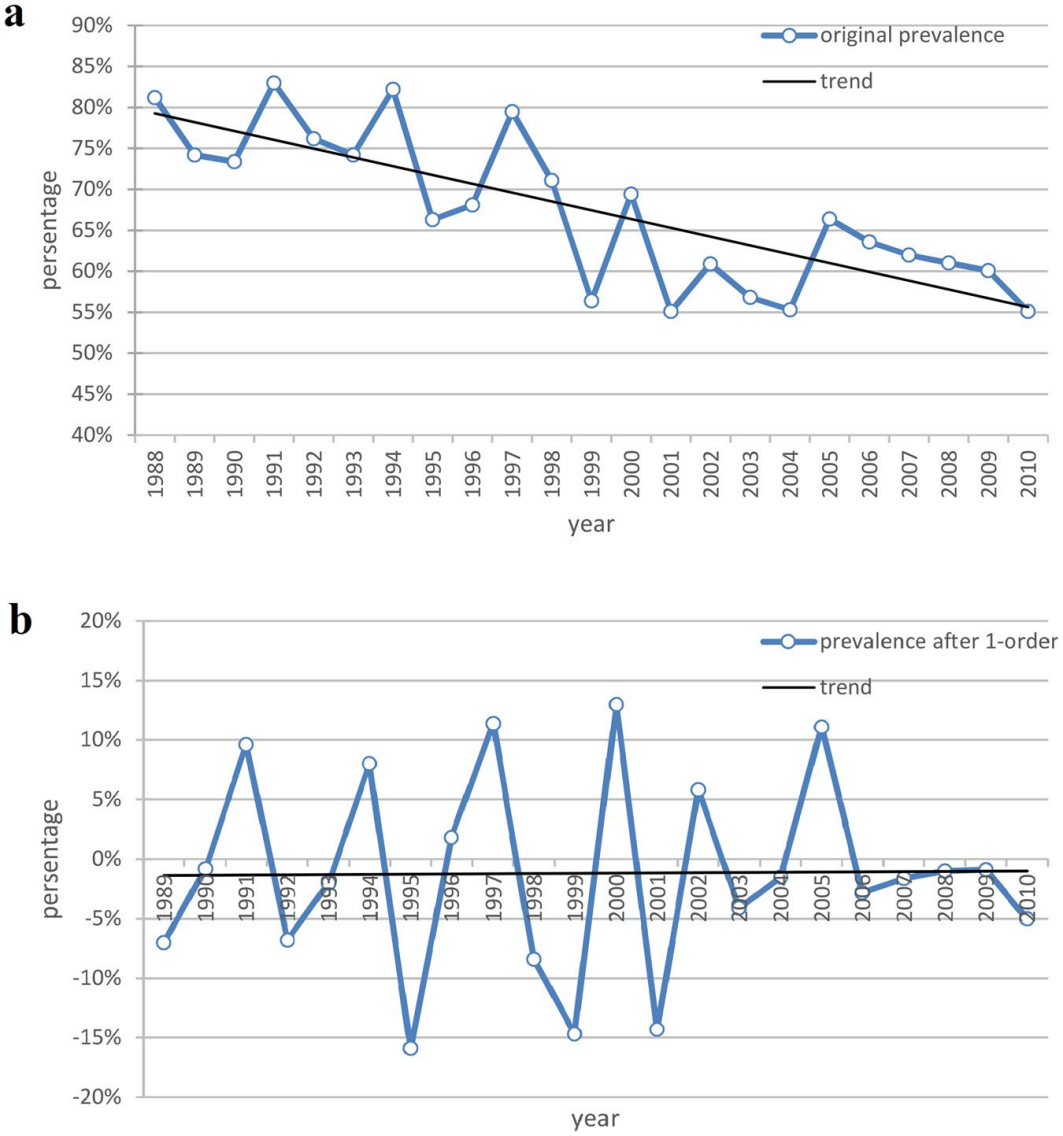
(**a**) Temporal trend of early childhood caries prevalence in China during 1988–2010; (**b**) 1-order differencing of ECC prevalence.

**Figure 4:**
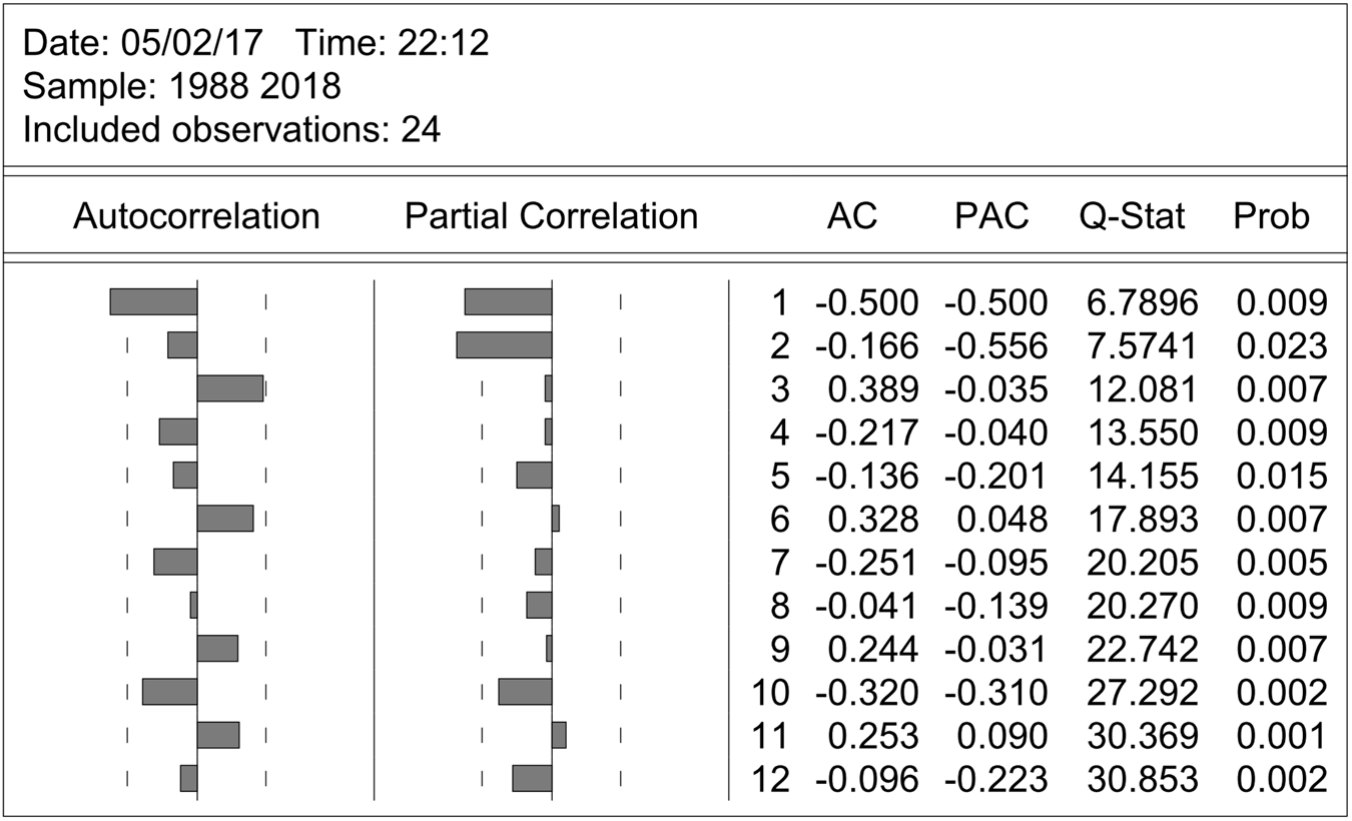
ACF, PACF and Q statistic of 1-order differencing sequence of ECC.

The prevalence rates fluctuated between 50% and 80% with a downward trend (Fig. 3a). The result of ADF test (*P* > 0.05) indicated that the sequence was non-stationary. After 1-order differencing was used, the differenced sequence tended stationary (Fig. 3b). The result of ADF test (*P* < 0.05) also showed that the sequence was stationary. Then, the figures of ACF and PACF were used to identify the parameter p and q (Fig. 4).

**Figure 5:**
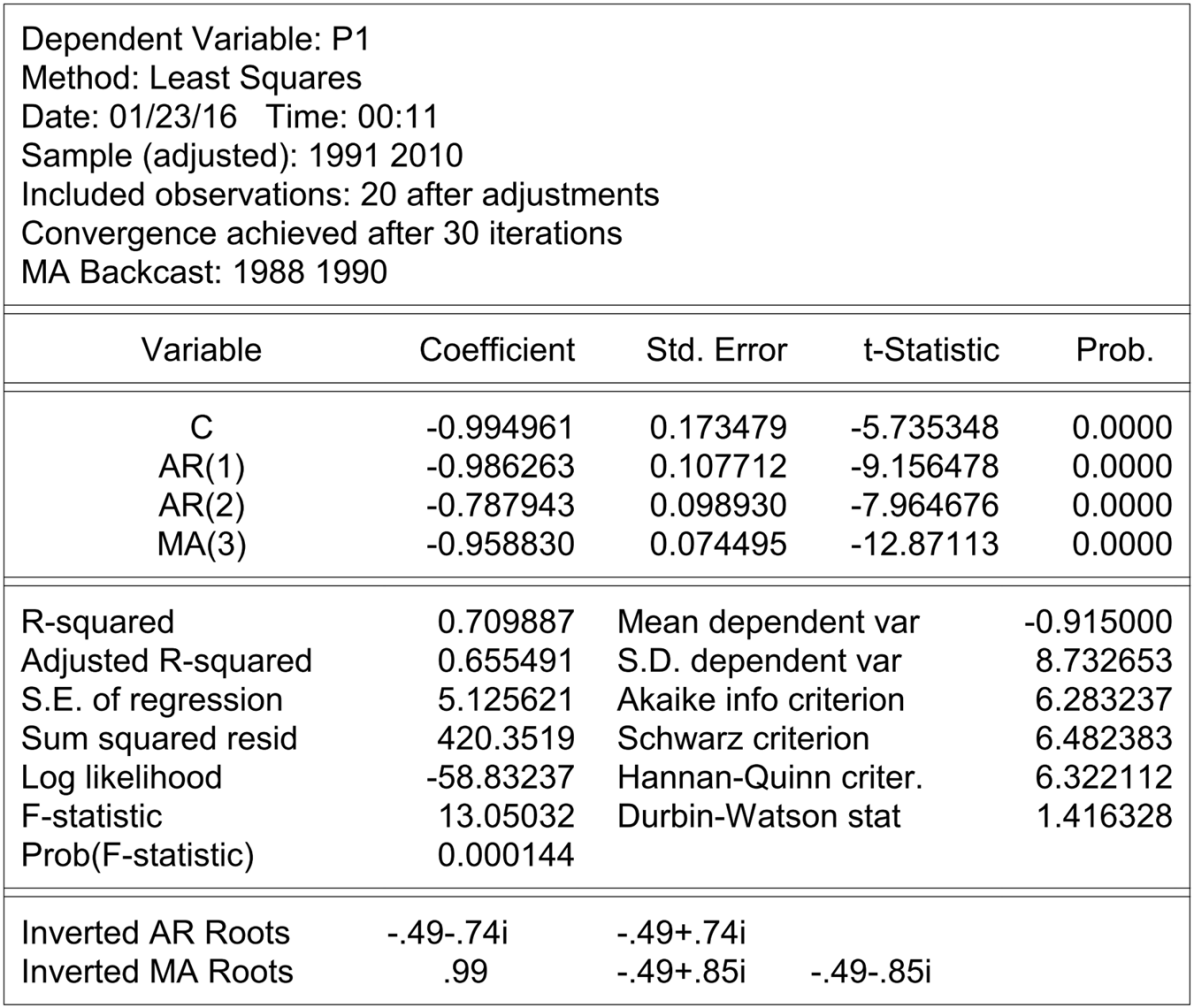
The result of AMIRA (2,1,3) model.

The ARIMA (2,1,3) was chosen. The fitted result showed the significance test of regression was 0.000144, which meant the equation was significant. Moreover, the AIC information was about 6.28 and the R-squared was about 0.71 (Fig. 5). These meant that the effect of the model was good.

Thus, the ARIMA (2,1,3) model on the original sequence has been established.

#### GM (1,1)

Firstly, we generated the accumulative sequence based on the original sequence, shown in Table 1. Then, using the method introduced above, the prediction model was as follows:

4 $$y(t)=-4988.19{e}^{-0.01587}+5069.39$$

**Table 1:** Result of accumulative sequence with GM.

Year	Original Xt (%)	Accumulative Yt (%)
1988	81.2	81.2
1989	74.2	155.4
1990	73.4	228.8
1991	83	311.8
1992	76.2	388
1993	74.2	462.2
1994	82.2	544.4
1995	66.3	610.7
1996	68.1	678.8
1997	79.5	758.3
1998	71.1	829.4
1999	56.4	885.8
2000	69.4	955.2
2001	55.1	1010.3
2002	60.9	1071.2
2003	56.8	1128
2004	55.3	1183.3
2005	66.4	1249.7
2006	63.6	1313.3
2007	62.0	1375.3
2008	61.0	1436.3
2009	60.1	1496.4
2010	55.1	1551.5

Thus, the GM (1,1) model was established.

#### Comparison of the results from fitting and prediction

The established ARIMA (2,1,3) model was compared with GM (1,1) from two aspects of fitting and prediction. The comparison in fitting was shown in Table 2. While the average MAE and MAPE of GM (1,1) were 4.81% and 7.34%; the average MAE and MAPE of ARIMA (2,1,3) were 3.63% and 5.74%. Therefore, ARIMA was better than GM in fitting performance.

**Table 2:** Fitting results of two models.

Year	Actual (%)	ARIMA (2,1,3)			GM (1,1)		
		Fitted (%)	MAE (%)	MAPE (%)	Fitted (%)	MAE (%)	MAPE (%)
1988	81.2	NA	NA	NA	NA	NA	NA
1989	74.2	NA	NA	NA	78.5	4.3	5.8
1990	73.4	NA	NA	NA	77.3	3.9	5.3
1991	83.0	85.7	2.7	3.3	76.1	6.9	8.3
1992	76.2	71.3	4.9	6.4	74.9	1.3	1.7
1993	74.2	69.7	4.5	6.1	73.7	0.5	0.7
1994	82.2	81.4	0.8	1.0	72.6	9.6	11.7
1995	66.3	68.4	2.1	3.2	71.4	5.1	7.7
1996	68.1	68.6	0.5	0.7	70.3	2.2	3.2
1997	79.5	75.3	4.2	5.3	69.2	10.3	13
1998	71.1	66.1	5.0	7.0	68.1	3.0	4.2
1999	56.4	68.2	11.8	20.9	67.0	10.6	18.8
2000	69.4	70.7	1.3	1.9	66.0	3.4	4.9
2001	55.1	60.6	5.5	10.0	64.9	9.8	17.8
2002	60.9	67.5	6.6	10.8	63.9	3.0	4.9
2003	56.8	64.9	8.1	14.3	62.9	6.1	10.7
2004	55.3	58.8	3.5	6.3	61.9	6.6	11.9
2005	66.4	63.5	2.9	4.4	60.9	5.5	8.3
2006	63.6	61.7	1.9	3.0	60.0	3.6	5.7
2007	62.0	58.2	3.8	6.1	59.0	3.0	4.8
2008	61.0	60.3	0.7	1.1	58.1	2.9	4.8
2009	60.1	58.6	1.5	2.5	57.2	2.9	4.8
2010	55.1	55.4	0.3	0.5	56.3	1.2	2.2
Average			3.63	5.74		4.8	7.4

**Figure 6:**
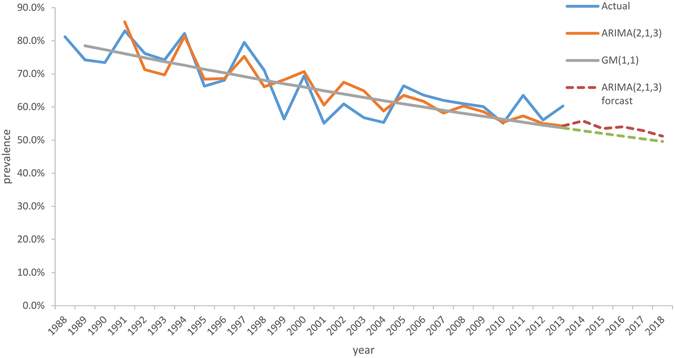
Two models’ fitting and prediction curves and the actual data curve.

In addition, the two models were used to predict the prevalence from 2011 to 2013, also showing that the ARIMA was better than GM with lower MAE and MAPE (Table 3). The fitting and prediction curves of two models were compared with actual curve (Fig. 6), indicating that ARIMA (2,1,3) model was more accurate and stable than the GM (1,1).

**Table 3:** Prediction results of two models.

Year	Actual value (%)	ARIMA (2,1,3)			GM (1,1)		
		Predicted value (%)	MAE (%)	MAPE (%)	Predicted value (%)	MAE (%)	MAPE (%)
2011	63.5	57.3	6.2	9.8	55.4	8.1	12.8
2012	56.1	55.0	1.1	2.0	54.5	1.6	2.9
2013	60.3	54.3	6.0	10.0	53.7	6.6	10.9
2014		55.8			52.8		
2015		53.5			52.0		
2016		54.0			51.2		
2017		52.9			50.4		
2018		51.2			49.6

## Discussion

Oral health in children is an important public issue in China and worldwide. Understanding the temporal trend of ECC may facilitate the allocation of oral health resource. As far as we know, this is the first study to forecast the trend of ECC in mainland China based on the data from meta-analysis. It has broadened a new method for the forecasting field, which may provide the base data and theoretical support to establish and evaluate the prevention measures of ECC. The time series data of prevalence of ECC were forecasted by two prediction models, both of which fitted for the data and can be used in forecasting. According to the fitting and prediction accuracy, ARIMA model outperforms the GM (1,1).

This study demonstrates that the prevalence of ECC has declined over the past 30 years and will continue to decrease in the future. The reasons for this declining trend could be the socioeconomic developments and improved public health service in China recently. Since its reform and opening-up, China has experienced rapid socioeconomic changes. The average annual economic growth rate was as high as 9.8%, and per capita gross domestic product increased from 1,112 RMB in 1987 to 38,420 RMB in 2012^25^. Governmental spending on public health care has grown greatly; the number of dentists has increased 13 times from 1985 to 2008^26, 27^; more and more oral health education programmes have been organized across the country^28^; parental awareness toward oral health and children’ oral habits have also been improved greatly according to the two national surveys^13, 14^. Our observation is consistent with many researches which have reported the inverse relationship between socioeconomic status and caries prevalence^29, 30^. If effective interventions are implemented in the near future, the prevalence of ECC may continue to decrease.

The fourth national oral health survey is in progress (2015–2016), and the related data (ECC prevalence at age 5) will be available in the near future. In this study, we have adopted ARIMA (2,1,3) model and GM (1,1) to forecast the ECC prevalence in 2014–2017, which is 53.5% and 52.0% in 2015, respectively. Acuracy of this prediction method based on data from meta-analyses will be further authenticated, compared with the results of the national survey. If the predicting result is close to the actual data, we can develop and popularize this method in the future research and application.

We have to mention several limitations in this study. First of all, the data used for forecasting were obtained from the pooled results of regional surveys. Error could not be avoided due to publication bias, sampling size and heterogeneity of the included articles^31^. Especially, pooling 1–6 years together included lots of variation and heterogeneities, and choosing year five was the appropriate time marker to demonstrate the epidemic trend for comparisons across studies. For the other age groups, it is not possible to set up the time series sequence due to limited literatures on other ages. Secondly, given the limited information of existing studies, we just conducted time series analysis without considering the risk factors which could affect the occurrence of ECC, such as medical expenditures, GDP, educational levels of the parents, and so on^4, 32, 33^. Further research can improve the efficacy of these models and provide more clues to explain the variation of the prevalence. Thirdly, there exist obvious economic and population differences among kinds of provinces and cities in China. However, we could not obtain sufficient annual or monthly ECC prevalence from the current publications, to construct the series analysis stratified by geographical and economic differences in different Chinese regions. Fourthly, we have noticed the constrained forecasting to extrapolate in our study, that is, the longer the forecasting duration, the lower the model’s accuracy^34, 35^. Finally, whether this new prediction method is suitable for other epidemic diseases needs further validation.

In general, the oral health status of children in China has improved over time. We have developed a new prediction method based on data from meta-analyses. Both ARIMA model and GM can be used in fitting and forecasting the prevalence of ECC in mainland China. More precise prediction models may be needed to explain the variation of the ECC trend. We aim to promote general awareness in the local Chinese governments to establish the epidemiological database of ECC on a regional level. Then, we can take the economic or population difference among kinds of provinces and cities in the further research, if we have sufficient information. Developing and applying these prediction models could make us better understand the epidemiological characteristics of ECC and be helpful to prevent and control this disease.

## Electronic supplementary material

Supplementary Information
