# Supplementary material for: Predicting trend of early childhood caries in mainland China: a combined meta-analytic and mathematical modelling approach based on epidemiological surveys
Source: Sci Rep. 2017 Jul 26;7:6507. doi: 10.1038/s41598-017-06626-w (PMC5529534; doi:10.1038/s41598-017-06626-w)
Supplement: Supplementary file 1 — Supplementary Information [file 41598_2017_6626_MOESM1_ESM.pdf]

Predicting trend of early childhood caries in mainland China: a combined meta-analytic and mathematical modelling approach based on epidemiological surveys

Xiaonan Zhang[1,2,3], Lei Zhang[4,5,6], Yonghong Zhang[7], Zhaoying Liao[8], Jinlin Song[1,2,3]

[1]College of Stomatology, Chongqing Medical University, Chongqing, China

[2]Chongqing key Laboratory of Oral Diseases and Biomedical Sciences, Chongqing, China

[3]Chongqing Municipal Key Laboratory of Oral Biomedical Engineering of Higher Education, Chongqing, China

[4]Melbourne Sexual Health Centre, Alfred Health, Melbourne, VIC, Australia.

[5]Central Clinical School, Faculty of Medicine, Nursing and Health Sciences, Monash University, Melbourne, VIC, Australia.

[6]Research Center for Public Health, School of Medicine, Tsinghua University, Beijing, China

[7]Medicine Engineering Research Center, College of Pharmacy, Chongqing Medical University, Chongqing, China

[8]Children's Hospital of Chongqing Medical University, Chongqing, China

Corresponding to:

Jinlin Song, College of Stomatology, Chongqing Medical University, Chongqing, China, No.426 Songshibei Road, Yubei District, Chongqing, China; Tel: +86 23 88860026; Fax: +86 23 89035721; E-mail: soongjl@163.com

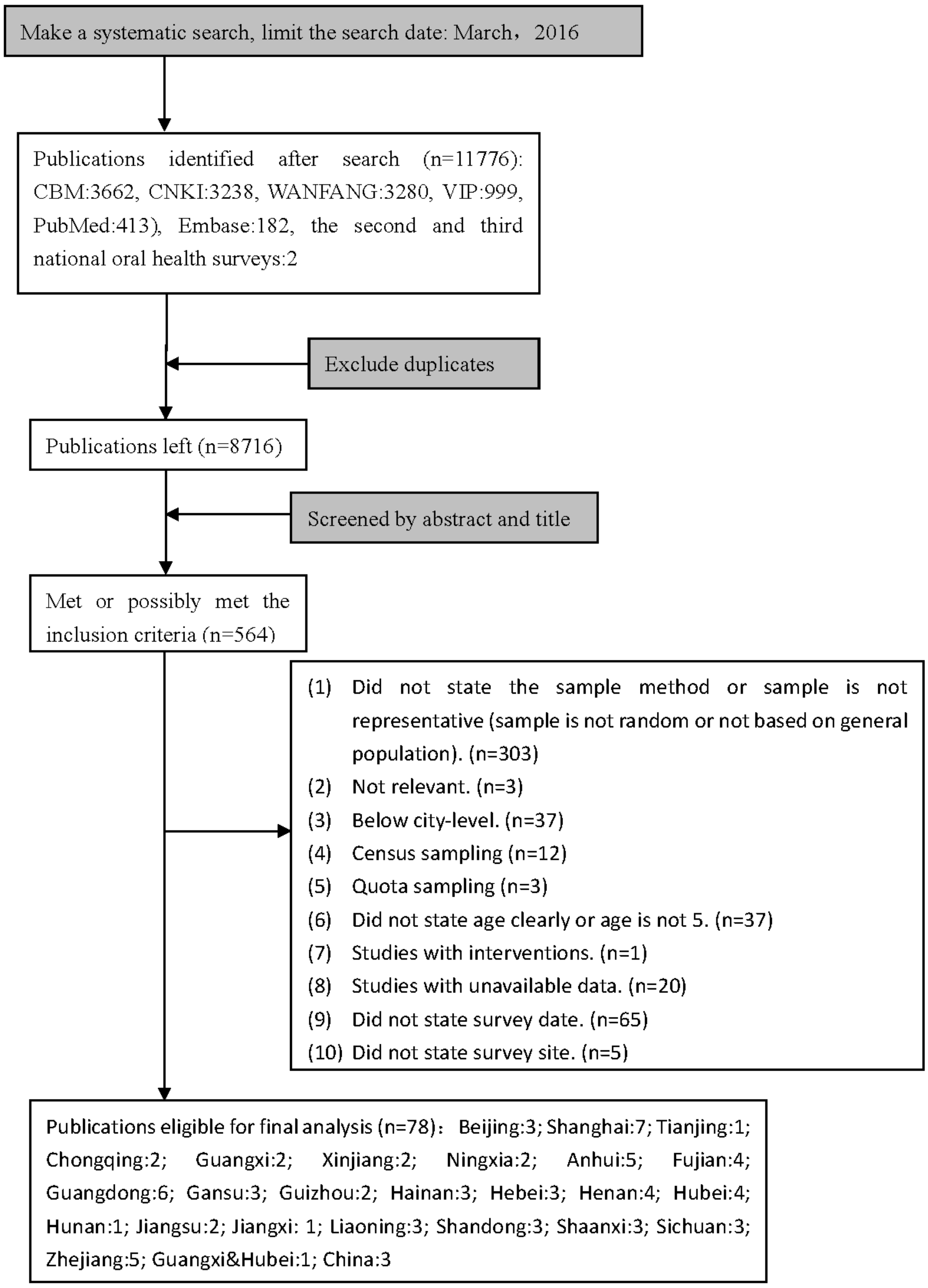

Fig. S1 Flow chart of literature search and selection

Table S1 | Characteristic of the included 78 studies

First author & published year	Survey date	Provinces	Territorial levels	U&R	Investigators	Sampling method	Diagnostic criteria	Sample size	Case size	Prevalence (%)	Quality score
Chen et al. 2008[1]	2007	Hainan	city	U	experienced child health doctors	random	WHO oral health survey	534	361	67.60	8
Chen.2009[2]	2007	Hainan	city	U	experienced child health doctors	random	WHO oral health survey	432	234	54.25	8
Chen et al. 1992[3]	1990	Guangdong	provincial	U&R	health care researchers and medical students	multistage random	WHO oral health survey	448	295	65.85	9
Cheng et al. 2006[4]	2005	Liaoning	city	NA	trained experienced dentists	random	Guideline for the 2nd National Oral Health Survey	1706	1364	79.95	9
Chen et al. 2008[5]	2006	Zhejiang	city	U	experienced doctors	cluster random	WHO oral health survey	405	353	87.16	8
Deng et al. 1998[6]	1995	Hunan	city	U	health care doctors	cluster random	NA	789	497	62.99	8
Dong et al. 2013[13]	2012	Shandong	city	U&R	NA	cluster	National uniform standards of dental caries	198	254	53.03	8
Du et al. 2007[7]	2001	Guangxi, Hubei	provincial	U&R	trained experienced dentists	stratified random	WHO oral health survey	806	504	62.53	9
Fan et al. 2010[8]	2008	Guangdong	city	U&R	NA	multi-stage stratified random	Guideline for the 3rd National Oral Health Survey	1440	808	56.11	10
Feng et al. 2004[9]	2000	Fujian	provincial	U&R	trained dental students	stratified cluster	WHO oral health survey	2132	1754	82.27	9

Feng et al. 2013[10]	2011	Shanghai	city	NA	dentists	cluster	WHO oral health survey	1015	526	51.82	8
Feng et al. 2013[10]	2003	Shanghai	city	NA	dentists	cluster	WHO oral health survey	812	530	65.27	8
Gao et al. 2009[11]	2007	Anhui	provincial	U&R	dentists	multi-stage stratified random	WHO oral health survey	431	283	65.66	9
Gao et al. 2010[12]	2008	Xinjiang	city	U	trained doctors	stratified cluster random	Guideline for the 2nd National Oral Health Survey	462	210	45.45	8
Gou et al. 2010[14]	2008	Chongqing	city	U	NA	cluster random	WHO oral health survey	1008	367	36.41	8
Hu et al. 1991[15]	1989	Henan	city	NA	NA	cluster	WHO oral health survey	31	23	74.19	8
Hu et al. 2008[16]	2007	Jiangsu	city	U	dentists	random	Guideline for the 2nd National Oral Health Survey	815	534	65.32	7
Hu.2013[17]	2012	Anhui	city	U	dentists	random	WHO oral health survey	1209	601	49.71	8
Jiang et al. 2002[18]	1999	Anhui	provincial	U&R	NA	multi-stage stratified random	Guideline for the 2nd National Oral Health Survey	2317	1523	65.73	10
Jiang et al. 2012[19]	2011	Shaanxi	city	U	trained pediatricians	cluster	WHO oral health survey	116	30	25.86	8
Le et al. 1992[20]	1992	Shanghai	city	NA	trained investigators	random	WHO oral health survey	336	256	76.19	8
Li et al. 1991[21]	1988	Hubei	city	NA	dentists	cluster	Criteria from <Oral Medicine>	344	282	81.98	8

Li et al. 1997[22]	1995	mainland	China	U&R	pediatricians	cluster random	WHO oral health survey	14393	6619	45.99	9
Li et al. 2007[23]	2006	Fujian	city	NA	NA	random	WHO oral health survey	1760	950	53.98	8
Li et al. 2011[24]	2008	Fujian	city	U&R	dentists	multi-stage stratified random	Guideline for the 3rd National Oral Health Survey	581	455	78.30	8
Li et al. 2011[25]	2009	Shanghai	city	U&R	NA	multi-stage stratified random	WHO oral health survey	1135	664	58.5	8
Li et al. 2012[26]	2008	Shanghai	provincial	U	dentists	cluster random	Guideline for the 3rd National Oral Health Survey	950	603	63.47	8
Li et al. 2012[26]	2009	Shanghai	provincial	U	dentists	cluster random	Guideline for the 3rd National Oral Health Survey	950	608	64.00	8
Li et al. 2012[26]	2010	Shanghai	provincial	U	dentists	cluster random	Guideline for the 3rd National Oral Health Survey	900	584	64.89	8
Li et al. 2012[26]	2011	Shanghai	provincial	U	dentists	cluster random	Guideline for the 3rd National Oral Health Survey	900	580	64.44	8
Li et al. 2013[27]	2011	Hebei	city	NA	dentists	stratified cluster	WHO oral health survey	303	230	75.91	8
Li et al. 2013[28]	2012	Shandong	city	U&R	experienced dentists	stratified random	Guideline for the 3rd National Oral Health Survey	426	270	63.38	8
Li. 2001[29]	1998	Jiangxi	city	NA	dentists	stratified cluster random	Guideline for the 2nd National Oral Health Survey	104	78	75.00	8
Li.2010[30]	2008	Gansu	city	U&R	health care doctors	stratified cluster random	Guideline for the 3rd National Oral Health Survey	400	244	61.0	8
Li. 2012[31]	2011	Tianjin	city	U	specialists from prevention station	random	Guideline for the 3rd National Oral Health Survey	269	205	76.21	9

Liang et al. 2010[32]	2009	Guangdong	city	U	dentists and health care doctors	cluster random	Guideline for the 2nd National Oral Health Survey	728	414	56.87	7
Liang et al. 2012[33]	2011	Guangdong	city	U	health care doctors	cluster random	Guideline for the 3rd National Oral Health Survey	1460	914	62.60	8
Lin et al. 2003[34]	2002	Chongqing	provincial	U&R	dentists	stratified cluster	WHO oral health survey	3261	1855	56.88	8
Liu et al. 2005[35]	1990	Ningxia	city	U	experienced dentists	random	WHO oral health survey	227	186	81.94	8
Liu et al. 2005[35]	1996	Ningxia	city	U	experienced dentists	random	WHO oral health survey	279	174	62.37	8
Liu et al. 2005[35]	2001	Ningxia	city	U	experienced dentists	random	WHO oral health survey	238	104	43.70	8
Liu et al. 2012[36]	2010	Ningxia	city	NA	NA	cluster random	WHO oral health survey	225	157	69.78	8
Liu et al. 2014[37]	2013	Hebei	city	NA	experienced dentists	multi-stage stratified cluster	WHO oral health survey	726	447	61.57	9
Liu et al. 2015[38]	2013	Beijing	province	U&R	dentists	randum	WHO oral health survey	107179	64629	60.30	9
Mao et al. 1997[39]	1995	Zhejiang	city	U&R	trained dentists	multi-stage stratified cluster	Guideline for the 2nd National Oral Health Survey	720	626	86.94	9
Mao et al. 2011[40]	2010	Sichuan	city	R	NA	cluster random	Criteria from <Practical internal medicine >	794	182	22.92	7
Meng et al. 2008[41]	2006	Beijing	city	R	dentists	stratified cluster random	National uniform standards of dental caries	230	96	41.74	8
NCOH.1998[42]	1995	Beijing	China	U&R	trained experienced dentists	multi-stage stratified cluster	Guideline for the 2nd National Oral Health Survey	2132	1611	75.56	10

NCOH.1998[42]	1995	Gansu	China	U&R	trained experienced dentists	multi-stage stratified cluster	Guideline for the 2nd National Oral Health Survey	2132	1711	80.25	10
NCOH.1998[42]	1995	Guangdong	China	U&R	trained experienced dentists	multi-stage stratified cluster	Guideline for the 2nd National Oral Health Survey	2132	1638	76.83	10
NCOH.1998[42]	1995	Hubei	China	U&R	trained experienced dentists	multi-stage stratified cluster	Guideline for the 2nd National Oral Health Survey	2132	1476	69.23	10
NCOH.1998[42]	1995	Liaoning	China	U&R	trained experienced dentists	multi-stage stratified cluster	Guideline for the 2nd National Oral Health Survey	2132	1908	89.49	10
NCOH.1998[42]	1995	Shandong	China	U&R	trained experienced dentists	multi-stage stratified cluster	Guideline for the 2nd National Oral Health Survey	2132	1607	75.38	10
NCOH.1998[42]	1995	Shanghai	China	U&R	trained experienced dentists	multi-stage stratified cluster	Guideline for the 2nd National Oral Health Survey	2132	1669	78.28	10
NCOH.1998[42]	1995	Sichuan	China	U&R	trained experienced dentists	multi-stage stratified cluster	Guideline for the 2nd National Oral Health Survey	2132	1292	60.6	10
NCOH.1998[42]	1995	Tianjin	China	U&R	trained experienced dentists	multi-stage stratified cluster	Guideline for the 2nd National Oral Health Survey	2132	1590	74.58	10
NCOH.1998[42]	1995	Zhejiang	China	U&R	trained experienced dentists	multi-stage stratified cluster	Guideline for the 2nd National Oral Health Survey	2132	1836	86.12	10
NCOH.1998[42]	1995	Yunnan	China	U&R	trained experienced dentists	multi-stage stratified cluster	Guideline for the 2nd National Oral Health Survey	2132	1615	75.75	10
NCOH.2009[43]	2005	Anhui	China	U&R	trained experienced dentists	multi-stage stratified random	Guideline for the 3rd National Oral Health Survey	795	510	64.15	10
NCOH.2009[43]	2005	Beijing	China	U&R	trained experienced dentists	multi-stage stratified random	Guideline for the 3rd National Oral Health Survey	791	464	58.66	10
NCOH.2009[43]	2005	Fujian	China	U&R	trained experienced dentists	multi-stage stratified random	Guideline for the 3rd National Oral Health Survey	790	599	75.82	10

NCOH.2009[43]	2005	Gansu	China	U&R	trained experienced dentists	multi-stage stratified random	Guideline for the 3rd National Oral Health Survey	785	438	55.8	10
NCOH.2009[43]	2005	Guangdong	China	U&R	trained experienced dentists	multi-stage stratified random	Guideline for the 3rd National Oral Health Survey	788	536	68.02	10
NCOH.2009[43]	2005	Guangxi	China	U&R	trained experienced dentists	multi-stage stratified random	Guideline for the 3rd National Oral Health Survey	782	652	83.38	10
NCOH.2009[43]	2005	Guizhou	China	U&R	trained experienced dentists	multi-stage stratified random	Guideline for the 3rd National Oral Health Survey	767	344	44.85	10
NCOH.2009[43]	2005	Hainan	China	U&R	trained experienced dentists	multi-stage stratified random	Guideline for the 3rd National Oral Health Survey	758	577	76.12	10
NCOH.2009[43]	2005	Hebei	China	U&R	trained experienced dentists	multi-stage stratified random	Guideline for the 3rd National Oral Health Survey	792	547	69.07	10
NCOH.2009[43]	2005	Henan	China	U&R	trained experienced dentists	multi-stage stratified random	Guideline for the 3rd National Oral Health Survey	784	465	59.31	10
NCOH.2009[43]	2005	Heilongjiang	China	U&R	trained experienced dentists	multi-stage stratified random	Guideline for the 3rd National Oral Health Survey	789	565	71.61	10
NCOH.2009[43]	2005	Hubei	China	U&R	trained experienced dentists	multi-stage stratified random	Guideline for the 3rd National Oral Health Survey	791	448	56.64	10
NCOH.2009[43]	2005	Hunan	China	U&R	trained experienced dentists	multi-stage stratified random	Guideline for the 3rd National Oral Health Survey	688	408	59.3	10
NCOH.2009[43]	2005	Jiangsu	China	U&R	trained experienced dentists	multi-stage stratified random	Guideline for the 3rd National Oral Health Survey	792	547	69.07	10
NCOH.2009[43]	2005	Jiangxi	China	U&R	trained experienced dentists	multi-stage stratified random	Guideline for the 3rd National Oral Health Survey	790	495	62.66	10
NCOH.2009[43]	2005	Liaoning	China	U&R	trained experienced dentists	multi-stage stratified random	Guideline for the 3rd National Oral Health Survey	791	585	73.96	10

NCOH.2009[43]	2005	Ningxia	China	U&R	trained experienced dentists	multi-stage stratified random	Guideline for the 3rd National Oral Health Survey	744	400	53.76	10
NCOH.2009[43]	2005	Shandong	China	U&R	trained experienced dentists	multi-stage stratified random	Guideline for the 3rd National Oral Health Survey	785	524	66.75	10
NCOH.2009[43]	2005	Shanxi	China	U&R	trained experienced dentists	multi-stage stratified random	Guideline for the 3rd National Oral Health Survey	792	513	64.77	10
NCOH.2009[43]	2005	Shaanxi	China	U&R	trained experienced dentists	multi-stage stratified random	Guideline for the 3rd National Oral Health Survey	787	354	44.98	10
NCOH.2009[43]	2005	Shanghai	China	U&R	trained experienced dentists	multi-stage stratified random	Guideline for the 3rd National Oral Health Survey	789	566	71.74	10
NCOH.2009[43]	2005	Sichuan	China	U&R	trained experienced dentists	multi-stage stratified random	Guideline for the 3rd National Oral Health Survey	780	458	58.72	10
NCOH.2009[43]	2005	Tianjin	China	U&R	trained experienced dentists	multi-stage stratified random	Guideline for the 3rd National Oral Health Survey	761	436	57.29	10
NCOH.2009[43]	2005	Xinjiang	China	U&R	trained experienced dentists	multi-stage stratified random	Guideline for the 3rd National Oral Health Survey	788	529	67.13	10
NCOH.2009[43]	2005	Zhejiang	China	U&R	trained experienced dentists	multi-stage stratified random	Guideline for the 3rd National Oral Health Survey	788	627	79.57	10
NCOH.2009[43]	2005	Chongqing	China	U&R	trained experienced dentists	multi-stage stratified random	Guideline for the 3rd National Oral Health Survey	748	480	64.17	10
NCOH.2009[43]	2005	Yunnan	China	U&R	trained experienced dentists	multi-stage stratified random	Guideline for the 3rd National Oral Health Survey	790	504	63.8	10
NCOH.2009[43]	2005	Neimenggu	China	U&R	trained experienced dentists	multi-stage stratified random	Guideline for the 3rd National Oral Health Survey	780	576	73.85	10
NCOH.2009[43]	2005	Jilin	China	U&R	trained experienced dentists	multi-stage stratified random	Guideline for the 3rd National Oral Health Survey	773	634	82.02	10

NCOH.2009[43]	2005	Qinghai	China	U&R	trained experienced dentists	multi-stage stratified random	Guideline for the 3rd National Oral Health Survey	787	562	71.41	10
Pei. 2008[44]	2007	Henan	city	NA	trained experienced dentists	cluster random	WHO oral health survey	397	273	68.77	8
Peng et al. 2001[45]	1997	Guangdong	city	NA	experienced dentists	multi-stage cluster random	NA	639	508	79.5	8
Quan. 1992[46]	1990	Hubei	city	U	NA	random	NA	120	87	72.50	9
Sheng.2008[47]	2007	Liaoning	city	NA	dentists	cluster	WHO oral health survey	202	126	62.38	8
Shu et al. 2000[48]	1996	Shanghai	city	NA	NA	cluster	Guideline for the 2nd National Oral Health Survey	50	36	72.00	8
Song et al. 2011[49]	2008	Shaanxi	city	NA	experienced dentists	cluster random	WHO oral health survey	143	77	53.85	8
Tao.2010[50]	2007	Anhui	city	U	trained health care doctors	stratified cluster random	Guideline for the 3rd National Oral Health Survey	1310	658	50.23	8
Tian et al. 2009[51]	2005	Hainan	provincial	U&R	trained experienced dentists	multiple stratified random	WHO oral health survey	720	548	76.11	9
Wang et al. 1998[52]	1996	Shandong	city	U	trained doctors	stratified random	WHO oral health survey	839	598	71.28	8
Wang et al. 2001[53]	1999	Shandong	city	U	doctors	cluster random	WHO oral health survey	391	192	49.10	8
Wang et al. 2010[54]	2008	Zhejiang	city	NA	trained doctors	cluster random	WHO oral health survey	928	484	52.16	8
Wang et al. 2011[55]	2008	Hebei	city	U	Health care doctors	random	WHO oral health survey	1672	1374	82.18	8

Wang et al. 2011[56]	2009	Zhejiang	city	U&R	trained dentists	cluster random	Guideline for the 3rd National Oral Health Survey	379	261	68.87	8
Wang.1989[57]	1988	Henan	city	U	NA	stratified cluster	criteria from <Oral Medicine>	126	106	84.13	8
Wei et al. 2013[58]	2012	Hubei	city	U&R	dentists	random	WHO oral health survey	149	61	40.94	8
Wen et al. 2013[59]	2011	Fujian	city	U&R	trained doctors	stratified cluster random	WHO oral health survey	720	575	79.86	8
Wu et al. 1995[60]	1993	Jiangsu	city	NA	NA	stratified random	criteria from "National Survey of Student Physical Health Research Handbook"	1058	785	74.20	8
Wu et al. 2005[61]	2002	Guangxi	city	NA	trained investigators	multi-stage stratified cluster random	WHO oral health survey	320	216	67.50	8
Xie et al. 2001[62]	2000	Anhui	city	U	NA	stratified random	WHO oral health survey	198	96	48.48	8
Xu.1995[63]	1994	Guandong	city	NA	NA	random	NA	276	231	83.69	7
Xu.2013[64]	2011	Sichuan	city	U&R	trained dentists	multistage stratified random	Guideline for the 3rd National Oral Health Survey	756	415	54.89	9
Ye.2010[65]	2008	Guangxi	city	U	dentists	random	NA	520	410	78.85	8
Ye et al. 2003[66]	1991	Hubei	city	U	dentists	cluster	WHO oral health survey	289	240	83.05	8
Ye et al. 2003[66]	1994	Hubei	city	U	dentists	cluster	WHO oral health survey	289	233	80.62	8

Ye et al. 2003[66]	1998	Hubei	city	U	dentists	cluster	WHO oral health survey	278	181	65.1	8
Ye et al. 2003[66]	2000	Hubei	city	U	dentists	cluster	WHO oral health survey	249	193	77.51	8
Yu et al. 2006[67]	2004	Guizhou	city	U&R	experienced dentists	Multi-stage stratified cluster	Guideline for the 2nd National Oral Health Survey	1100	608	55.27	10
Yu.2010[68]	2009	Zhejiang	city	U	experienced dentists	random	Guideline for the 3rd National Oral Health Survey	320	166	51.88	8
Zhang et al. 2000[69]	1995	Shanghai	provincial	U	trained investigators	cluster random	WHO oral health survey	1599	945	59.10	8
Zhang et al. 2004[70]	2003	Shanghai	provincial	U&R	health care doctors	random	WHO oral health survey	684	310	45.32	8
Zhang et al. 2005[71]	2000	Liaoning	city	NA	dentists	random	WHO oral health survey	579	396	68.39	8
Zhang et al. 2011[72]	2008	Shaanxi	city	U	experienced dentists and health care doctors	random	WHO oral health survey	608	391	64.31	8
Zhao.2000[73]	1999	Gansu	city	U	NA	random	Guideline for the 2nd National Oral Health Survey	540	444	82.22	8
Zhao.2001[74]	1998	Gansu	provincial	U&R	trained experienced dentists	random	Guideline for the 2nd National Oral Health Survey	1222	929	76.02	8
Zhong et al. 2003[75]	2001	Henan	provincial	U&R	experienced dentists	multi-stage stratified cluster	WHO oral health survey	2320	1326	57.16	9
Zhou et al. 2004[76]	2002	Beijing	city	U	NA	stratified cluster random	Guideline for the 2nd National Oral Health Survey	620	369	59.52	8

Zhu.2003[77]	1999	Sichuan	city	R	trained dentists	random	WHO oral health survey	1470	419	28.50	8
Zhu.2012[78]	2011	Beijing	city	U	experienced dentists	random	Guideline for the 3rd National Oral Health Survey	2400	1841	76.71	8

NCOH: National Committee for Oral Health; NA: not available; U: urban; R: rural; WHO: World Health Organization; AADA: American Academy of Pediatric Dentistry

A total score of 10 represents the lowest risk of bias and 0 represents the highest risk of bias.

References

1 Chen, C. B., Chen, H. Q., Xing, C. Q. Caries survey report of 2354 2~6 year- old chidren in Sanya. *Maternal Child Health Care Chin* **23**, 4330-4332 (2008).

2 Chen, H. Q. 1866 preschool children's prevalence of dental caries in Sanya. *Maternal Child Health Care Chin* **20**, 2825-2826 (2009).

3 Chen, S. X., Zhang, Y., Song, S. D., Zhang, Y. N., Ou, Y. The comprison of people's oral health between rural and urban areas. *J Guangdong Pharm Univ* **8**, 39-43 (1992).

4 Cheng, R. B., Zhang, X. F., Zhang, Y., Pan, L., Tao, W. An epidemiological investigation of deciduous dental caries among 5375 preschool children aged between 3 to 6 years in Shenyang city. *Shanghai J Stomatol* **15**, 596-600 (2006).

5 Chen, M., Peng, S., Zheng, J., Jiang, J. L. Survey of caries and distribution for 1160 children from 4 to 6 years old in Ningbo city. *Chin Mod Doctor* **46**, 58,112 (2008).

6 Deng, Z. F., Guo, J. L., Xu, S. S. Investigation of 2956 children's caries in Changsha. *Pract Prevent Med* **5**, 241-242 (1998).

7 Du, M., Luo, Y., Zeng, X., Alkhatib, N., Bedi, R. Caries in preschool children and its risk factors in 2 provinces in China. *Quintessence Int* **38**, 143-151 (2007).

8 Fan, W. H., Huang, S. H., Zheng, Z., Chen, W. C., Zhang, H. C. 2008 Guangzhou children deciduous caries epidemiological survey. *J Dent Prevent Treat* **18**, 127-130 (2010).

9 Feng, Y. *et al.* Fujian 5-year-old children's oral health epidemiological survey report. *J Fujian Med Univ* **38**, 87-90 (2004).

10 Feng, H., Cai, W. 5 year-old children's caries survey report in Changning district of Shanghai. *Shanghai J Prevent Med* **25**, 99-100 (2013).

11 Gao, Y. M., Han, X. L., Yan, Y. C. The current situation of five-year-old children's caries and thier parents' oral ideas. *Acta Univ Med Anhui* **44**, 120-123 (2009).

12 Gao, X., Xu, Y. H., XU, P. R. Survey on health status of preschool children in Urumqi. *Maternal Child Health Care Chin* **21**, 3007-3009 (2010).

13 Dong, G. X., Gao, M., Ma, C. Survey report of preschool children'scaries in Laiwu. *J Commun Med* **11**, 56-57 (2013).

14 Gou, Q. Y., Zhou, Q., Zhou, W. L., Jiang, X. Q., Liu, L. Survey on dental caries among 3804 preschool children in Chongqing. *Maternal Child Health Care Chin* **25**, 2854-2855 (2010).

15 Hu, Q. Y. *et al.* Caries survey report of 1773 children in Zhengzhou. *J Henan med Univ* **26**, 371-373 (1991).

16 Hu, Q. G., Ren, C. Kingdergarden's children's oral health survey result in Wuxi. *Chin J Pract Stomatol* **1**, 249 (2008).

17 Hu, D. X. Analysis on dental caries of preschool children in Tongling. *Chin J General Pract* **4**, 598-599 (2013).

18 Jiang, Y., Han, X. L., Zhang, L., Yan, Y. C. Sampling and cause analysis of five-year age groups decidudious caries prevalence and treatment needs in Anhui. *Acta Univ Med Anhui* **37**, 237-240 (2002).

19 Jiang, H., Wang, X. Y. Invastigation and analysis of children's caries and oral health in Weinan. *Chin J Child Health Care* **20**, 474-475 (2012).

20 Le, S. X. *et al.* 1112 chilren's oral health report in Shanghai. *Stomatol* **12**, 172-174 (1992).

21 Li, Y. L., Huang, J. M. Investigation of deciduous dental caries in preschool children in Shashi.

Hubei J Preven Med **2**, 13-14,48 (1991).
22 Ji, H., Yan, G. F. & Zhang, X. A comparative study on the incidence of dental caries of children 0~7 years in the urban and suburban rural areas of 9 cities of China. *Chin J Child Health Care* **5**, 213-215 (1997).
23 Li, R. Z., Su, R. Z. & He, Q. The investigation of caries of 1760 children aged from 2 to 7 years old in Xiamen. *Sci travel Med* **13**, 41-42 (2007).
24 Li, Y. *et al.* Associations of social and behavioural factors with early childhood caries in Xiamen city in China. *International journal of paediatric dentistry / the British Paedodontic Society [and] the International Association of Dentistry for Children* **21**, 103-111, doi:10.1111/j.1365-263X.2010.01093.x (2011).
25 Li, Y. L., du, Q. & Mao, X. Y. The epidemiological investigation into dental caries among the 5 years old in Shanghai suburban area. *Chin J Pract Stomatol* **4**, 413-415 (2011).
26 Li, C. R., Zeng, X. L., Wang, X., Xu, W., Chen, X. Analysis of caries status among 5-year-old children in Shanghai from 2008 to 2011. J *Shanghai stomatol* **21**, 451-454 (2012).
27 Li, Y., Wang, L., Dong, Q., Chen, H. W. 3~6 year-old children's caries epidemiological analysis in Tangshan. J *Heibei Med* **35**, 922 (2013).
28 Li, H., Li, A. Z., Wang, J. X., Jiang, Y. F. The present situation of five-year-old children's caries in Linyi city and its relevant factors. *J Shandong Med College* **35**, 444-447 (2013).
29 Li, X. Q. 1234 children's caries survey report in Nanchang city. *Mod Diagn Treat* **S0**, 40 (2001).
30 Li, X. Q. Dental caries among children aged 4 to 6 investigation and analysis in Dingxi city. *Chin Pediatrics Integr Tradit West Medi* **2**, 191-192 (2010).
31 Li, H. 3-5 years old children caries epidemiological analysis. J *Harbin Medi* **32**, 32 (2012).
32 Liang, M. P., Li, M. Q., Li, W. Z., Chen, X. N., Jiang, Y. Investigation and analysis of caries in Maoming kindergarten children aged 3-5. *Guide Chin Med* **8**, 71-72 (2010).
33 Liang, C. M., Zhao, X. G., Rong, L. H., Feng, C. Z., Li, J. F. Analysis of preschool children's caries in Jiangmen district. *Chin J Child Health Care* **20**, 761-762 (2012).
34 Lin, J. H. *et al.* Dental caries in children and adolescents of Chongqing. *J Chongqing Med Univ* **28**, 775-777,786 (2003).
35 Lu, Y., Chen, D. M., Jiang, Y., Huang, H. R. Study of dental caries prevalence in children of Yinchuan in China. *West Chin J stomatol* **23**, 502-504 (2005).
36 Liu, Y., Wu, M., Zhang, X. L., Duan, X. J. Yinchuan 5-ear-ld children deciduous teeth caries disease and related influence factors of investigation and analysis. J *Harbin Med* **32**, 421-424 (2012).
37 Liu, M., Gao P, Sun P., Yong, C. Epidemiological survey of early childhood for 5 year-old children in Langfang． Hebei in 2013. *Natl Med J Chin* **94**, 2201-2203 (2014).
38 Liu, M., Shi, Y. G., Peng, W. A survey of the early childhood caries in 3-5 years children in Beijing 2013, *Beijing J Stomatol* **23** (2015).
39 Mao, L. B. *et al.* Ningbo urban and rural residents caries epidemiology. *Mod Pract Med* **9**, 4-6 (1997).
40 Mao, K. Q., Yang, Y. B. Sample survey on health of children aged 0~6 years old in Xichang city. *Maternal Child Health Care Chin* **26**, 4852-4854 (2011).
41 Meng, J. *et al.* Oral examination quality analysis of Beijing kindergarten children in rural areas *Maternal Child Health Care China* **23**, 236-237 (2008).
42 Group, N. D. P. S. *Second national epidemiological survey of oral health*. 1 edn, (People's

Medical Publishing House, 1998).
43 Group, N. D. P. S. Third national epidemiological survey of oral health. (Beijing, 2008).
44 Pei, R. Survey of preschool children's oral health in Zhengzhou. *J Med Forum* **29**, 34-35 (2008).
45 Pen, J. *et al.* Epidemiological festures of dental caries among children and adolescent in Shenzhen. *Chin Trop Med* **1**, 263-264 (2001).
46 Quan, X. H., Liu, K. J. Investigation and analysis of caries among 598 children aged 2 to 5. *J Mod Stomatol* **6**, 162-163 (1992).
47 Sheng, A. 4-6 years old children's oral health survey and discuss prevention strategies in Benxi City. *Mod Med Health* **24**, 1568-1569 (2008).
48 Shu, C. B., Feng, X. P., Pan, Y. Investigation of deciduous teeth caries in children under 14-year-old. J *Shanghai stomatol* **9**, 175-177 (2000).
49 Song, K. M., Yu, G. X., Zhang, F., Li, J. J. & you, S. X. Survey of dental caries incidence in 412 children aged three to five years in Xi'an city. *Chin J Conserv Dent* **21**, 101-102,109 (2011).
50 M., T. G. 5 years old Children's deciduous caries Investigation and Analysis in Tongling. *J Tongling Vocational Technical College* **1**, 4-5 (2010).
51 Tian, Y. G., Liao, T. A., Xie, Q., Zhao, X. L., Xing, K. C. Caries prevalence among residents of Hainan province. *Chin Public Health* **25**, 1125-1126 (2009).
52 Wang, W. Y., Fu, P., Yu, S. Y. Caries investigation of preschool children in Qingdao. *Literature Inf Prevent Med* **4**, 193 (1998).
53 Wang, W. Y., Li, X. H., He, Y. F. Caries survey report of preschool children in Qingdao. *Chin J Healthy Birth Child Care* **12**, 139-140 (2001).
54 Wang, W. Q., Jing, C., Wang, H. H. Dental caries and oral health behavior of 5-year-old children in Hangzhou city. *Stomatol* **30**, 749-750 (2010).
55 Wang, Q. C., Wang, C. X. 2~6 year-old preschool children's caries survey report in Tangshan. *Maternal Child Health Care Chin* **26**, 3119-3120 (2011).
56 Wang, S. H., Zhu, S., Chen, J. P., Zhang, X., Jiang, F. An analysis of correlative factors of deciduous teeth caries in preschool children. *Zhejiang Prev Med* **23**, 5-8 (2011).
57 Wang, X. Y., Shi, A. M. Caries survey among children in Zhengzhou aged 3 to 6. *J Appl Clin Pediatrics* **4**, 368-371 (1989).
58 Wei, Y. H., Wang, F., Xu, W. Investigation of preschool children's caries. *J Clin Stomatol* **29**, 681-682 (2013).
59 Wen, Y. J. *et al.* Analysis of five-year-olds caries status and related factors in Ningde city of Fujian Province. *Chin J Prim Med and Pharm* **20**, 3589-3591 (2013).
60 Wu, S. Y., Yu, J., Xu, D. N. Epidemiological survey of 10491 children's oral health in Changzhou city. *Stomatol* **15**, 100-101 (1995).
61 W., W., Zeng, X. J., Huang, H. Survey reasult of 3~5 year-old children's caries in Nanning, Guangxi province. *J Mod Stomatol* **19**, 407 (2005).
62 Xie, X. M. 567 preschool children aged 3 to 7 deciduous caries survey. *Acta Univ Med Anhui* **36**, 321 (2001).
63 Xu, Y. C. 1051 preschool children's caries survey result in Jieyang city. *J Dent Prevent Treat* **3**, 54 (1995).
64 Xu, B. Q., Wen, X. Y., Guo, H. J. Investigation of dental caries of 5-year-old children and their parents' oral hygienic knowledge and behavior in Mianyang. *Maternal Child Health Care Chin*

23, 3815-3817 (2013).
65 Ye, Y. Survey reault of 3~6 year-old children's caries in Guigang, Guangxi province. *Natl Medi Frontiers Chin* **5**, 96, doi:10.3969/j.issn.1673-5552.2010.11.0072 (2010).
66 Ye, Y., Li, P., Li, X. T. Shiyan City caries in preschool children 10 years of observation. *Chin J Child Health Care* **11**, 141-142 (2003).
67 Yu, X. Y., Ma, L. X. Guiyang 5-year-old child deciduous caries sample survey report. *J Guizhou Med* **30**, 659-660 (2006).
68 Yu, J. 3~6 year- old children's caries survey result in Hangzhou. *Zhejiang Prev Med* **22**, 67,70 (2010).
69 Zhang, J., Wei, M., Zhang, Y. Caries investigation of children in Shanghai below 7 and its trend in 10 years. *Shanghai J Prev Med* **12**, 385-386 (2000).
70 Zhang, J. M., Zhu, J. F., Lu, Y. P. Investigation and treatment of dental caries in preschool children. *Anthol Med* **23**, 428-430 (2004).
71 Zhang, X. F., Xu, X. B., Cheng, R. B., Pan, L. [Study of caries polarization in 2-5 year-old children of Shenyang, China]. *West Chin J stomatol* **23**, 258-259 (2005).
72 Zhang, P., Yang, T. F., Li, Y. Q. 2626 children aged 2 to 6 's caries survey in Weinan. *J Shaanxi Med* **40**, 1372-1373 (2011).
73 Zhao, W. H. 5-year-old child deciduous caries sample survey report in Lanzhou. *Gansu Sci Technol* **4**, 48 (2000).
74 Zhao, W. H. Caries investigation and analysis of urban and rural, poor areas in Gansu Province. *Chin J Conserv Dent* **11**, 121-122 (2001).
75 Zhong, Y., Yang, B. S., He, J. Investigation of deciduous caries in preschool children in Henan. *Henan J prev Med* **14**, 141-143 (2003).
76 Zhou, H. L. *et al.* Statistics and analysis of Xicheng oral health survey. *J Beijing J Stomatol* **12**, 160-162 (2004).
77 Zhu, Q. H. Caries investigation and analysis of rural children in Bazhong. *Chin General Pract* **6**, 137-138 (2003).
78 Zhu, Z. G. Caries survey analysis of preschool children in Changping area, Beijing City, 2011. *J Hunan Univ Chin Med* **32**, 67-68 (2012).
